# Effects of a workplace exercise intervention on cardiometabolic health: a randomized controlled trial

**DOI:** 10.1186/s12889-025-24815-5

**Published:** 2025-10-15

**Authors:** Ali Muneer AlRahma, Mansoor Anwar Habib, Emad Masuadi, Tom Loney, Thomas Boillat, Syed Mahboob Shah, Luai A. Ahmed, Javaid Nauman

**Affiliations:** 1 Institute of Public Health, College of Medicine & Health Sciences, United Arab Emirates University, Al-Ain, United Arab Emirates; 2 Public Health Protection Department, Dubai Health Authority, Dubai, United Arab Emirates; 3 Emirates Integrated Telecommunications Company, Dubai, United Arab Emirates; 4 College of Medicine, Mohammed Bin Rashid University of Medicine and Health Sciences, Dubai Health, Dubai, United Arab Emirates; 5 Design Lab, College of Medicine, Mohammed Bin Rashid University of Medicine and Health Sciences, Dubai Health, Dubai, United Arab Emirates; 6 Department of Circulation and Medical Imaging, Faculty of Medicine and Health Sciences, Norwegian University of Science and Technology, Trondheim, Norway; 7 Healthy Living for Pandemic Event Protection (HL – PIVOT) Network, Chicago, IL USA

**Keywords:** Workplace exercise, Physical activity, Randomized controlled trial, Cardiovascular diseases, Cardiometabolic risk factors, And heart disease risk factors

## Abstract

**Background:**

The United Arab Emirates reports one of the highest mortality rates due to non-communicable diseases, and insufficient physical activity is a major underlying cause. The workplace environment can be an important setting to promote physical activity and overall health.

**Methods:**

We conducted a parallel, single-blinded, randomized controlled trial, enrolling adults (aged 18–59 years) from a semi-government telecommunications company with a waist circumference of ≥ 94 cm (≥ 90 cm for South and East Asians) for males and ≥ 80 cm for females. Eligible participants (*n* = 130) were randomly assigned (1:1) to the intervention group and delayed-intervention group (controls). The intervention group received two hours of weekly supervised exercises during working hours for 12 weeks. At the end of 12 weeks, the delayed-intervention group received two hours of weekly exercise for four weeks. The primary outcome was the change from baseline to week 12 in cardio-metabolic risk factors, including waist circumference, blood pressure, HDL cholesterol, triglycerides, and fasting glucose. The secondary outcome, assessed for the intervention group only, was the change in objectively measured physical activity from baseline to week 16. We used an intention-to-treat analytical approach.

**Results:**

Mean age of participants at enrolment was 36.9 years, and 25% were female. A total of 105 (81%) completed the 12-week follow-up measurements. We found no statistically significant change between groups from baseline to the end of the 12-week for primary outcome. However, the within-group change in the intervention group at week 12 was statistically significant for fasting plasma glucose [− 3.6 mg/dL (95% CI, − 6.7 to − 0.42)], HDL cholesterol [2.6 mg/dL (1.1 to 4.0)], and waist circumference [− 4.8 cm (− 6.1 to − 3.5)]. For the secondary outcome, overall physical activity levels increased with a significant increase in the estimated weekly average of vigorous-intensity physical activity [12.5 min (4.1 to 21.0)] at four weeks post intervention.

**Conclusions:**

We found no statistically significant difference between groups for primary outcomes. However, allocating time for exercise during working hours resulted in increased levels of physical activity and improved cardio-metabolic risk factors. Our study addresses a critical public health issue related to workers’ health in an office setting, highlighting the potential efficacy of implementing health-promoting strategies within the workplace environment to enhance employees’ health.

**Trial registration:**

ClinicalTrials.gov NCT04403789.

**Supplementary Information:**

The online version contains supplementary material available at 10.1186/s12889-025-24815-5.

## Introduction

Overwhelming evidence shows that insufficient physical activity (PA) is associated with chronic diseases and contributes to financial burden on health systems [1–7]. In contrast, regular PA may serve as an effective and cost-effective therapy that improves overall health [5, 6, 8, 9]. The rising levels of physical inactivity in the Eastern Mediterranean region (43%) and in the United Arab Emirates (38%) are alarming, and are comparable with the global levels of insufficient PA (31%) [10, 11]. The World Health Organization (WHO) 2018–2030 Global Action Plan on Physical Activity considers the workplace environment a vital arena that could promote PA and overall health [12, 13].

However, evidence for the effectiveness of PA interventions in workplace settings remains mixed. Recent workplace PA interventions have reported either a favourable outcome or no significant between-group differences for body composition and metabolic functions [14–19]. The findings of a meta-analysis showed improvement in PA through mobile health interventions, however, no significant difference in weight loss was reported in the intervention group compared with the control group [16]. Another systematic review and meta-analysis reported significant improvements in body weight, body mass index, and waist circumference associated with workplace PA interventions, however, no significant improvements were observed for blood pressure, lipids and blood glucose [19]. A recent umbrella review with meta-analysis and narrative synthesis reported a modest effect of workplace interventions on PA and sedentary behaviour, however, the quality of most of the included reviews was low or critical low with high levels of heterogeneity [20]. Therefore, well-designed studies with objective monitoring are still needed to determine the efficacy of practical workplace exercise interventions for improving cardiometabolic health, particularly in regions with high inactivity levels like the United Arab Emirates.

The European Network for Workplace Health Promotion (ENWHP) recommends a set of criteria for the promotion of PA in the workplace. The criteria include implementing approaches that encourage PA during working hours, weekends, and non-working hours. The ENWHP criteria also recommends providing easily accessible PA facilities and programmes in the workplace or at least in external sports facilities [21]. In addition, the Social-Ecological Model (SEM) identifies organizational and behavioural relationships for health promotion interventions within an organization. There are five levels of the SEM. They include the following: (1) individual level, (2) interpersonal level, (3) community level, (4) organizational level, and the (5) policy/enabling environment level [22]. Therefore, the most effective public health prevention and control approach should use a combination of the recommendations above.

We aimed to conduct a randomized clinical trial following the ENWHP recommendations and SEM aspects [21, 22]. The primary objective was to examine the effects of a workplace exercise intervention on cardio-metabolic health where the cardio-metabolic risk factors include waist circumference, blood pressure, high-density lipoprotein (HDL) cholesterol, triglycerides, and fasting plasma glucose. The secondary objective was to determine whether the workplace exercise intervention can improve PA levels four weeks post-intervention.

## Methods

### Study design

The study was a pragmatic, parallel, randomized controlled trial with a 1:1 allocation ratio to the intervention group (IN) and delayed intervention (DI) group. The trial is registered at ClinicalTrials.gov (NCT04403789) and was approved by the Dubai Scientific Research Ethics Committee in Dubai Health Authority (DSREC-SR-08/2019_02). The trial protocol including details on setting, randomization, allocation concealment, statistical analysis plan, and outcomes, was published previously [23]. A brief account of study procedure is described below.

## Participant recruitment

The recruitment was conducted as previously outlined in the published study protocol [23]. Briefly, all employees at the participating company received an email invitation to attend an information session. These sessions detailed the study objectives, eligibility criteria, and the intervention schedule, and emphasised the voluntary nature of participation. Interested employees provided written informed consent. Eligible consenting participants then underwent baseline health measurements at the worksite health centre. Initial recruitment rates were lower than anticipated. To improve engagement, we offered a small, relevant non-monetary incentive (gift vouchers of sports shop) upon completion of the final follow-up assessment. This strategy was successful in improving participant retention and enrolment numbers.

## Inclusion and exclusion criteria

Eligible adult participants aged 18 to 59 years with a waist circumference of ≥ 94 cm (≥ 90 cm for South and East Asians) for males and ≥ 80 cm for females were enrolled in the trial. The participants were required to be an employee in the telecommunications company, available for the study duration, willing to commit until the end of the intervention period, and must sign the consent sheet. Exclusion criteria included severe injury in the back or joints, a medical condition that could prevent them from exercising, advice from a physician not to exercise, pregnancy, planned major surgery during the study period, self-reported lung disease, cardiovascular disease, cancer, and current participation in other health promotion programs.

## Randomization and masking

The randomization sequence was computer generated and was stratified by sex and age using random block sizes of four and six by an independent statistician. The IN group was renamed Group A and the DI group as Group B, and participants were strongly urged not to reveal their allocation status.

## Intervention

The IN group received two hours of weekly exercise time during the normal working hours for 12 weeks. The two hours were used on two different days in a week (e.g., a maximum of one hour per day), either in the middle or at the end of their working hours. The 12-week intervention duration was selected based on recommendations of previous studies, demonstrating that this timeframe is effective to observe significant improvements in cardiometabolic outcomes [24–26]. The one-hour supervised exercise sessions were conducted in the workplace, following the recommendations of American College of Sports Medicine, targeting all major muscle groups at a moderate-to-high intensity exercise [27]. Certified trainers tailored the intensity to individual fitness levels by providing scaled exercise alternatives (e.g., adjusting resistance, range of motion, or exercise complexity) and supervising a personalized pace to ensure the exercise intensity was both safe and effective for each participant. The session started with five minutes of warm-up exercises, then 50 min of aerobic and resistance exercises, and ended with five minutes of cool-down period. The aerobic and resistance exercises were performed in 7–10 min bouts throughout the session. Further details of the intervention are available in the supplemental material and the published protocol [23].

The DI group was asked to maintain their usual lifestyle, and when the 12-week intervention period ended, they received two hours of exercise time per week from working hours for four weeks. However, exercise trainers did not supervise the sessions. The main purpose of providing exercise time to the DI group after the end of the intervention was to encourage them to participate in the study irrespective of their allocation.

### Measurements

Trained nurses in the workplace’s health centre who were blinded to group allocation conducted the anthropometric and clinical measurements, administered questionnaires, and performed the phlebotomy. Waist circumference was measured in centimetres using a measurement tape above the participants’ hipbones in a standing position. The measurement was taken when the tape was not compressed on the skin and after breathing out. Participants sat for at least 5 min on a chair with back support and resting diastolic and systolic blood pressure were measured through automated oscillometry. Finally, for the remaining risk factors, a butterfly needle was used to collect blood samples after 12-hours of fasting. The drawn blood samples were then stored in a − 20 °C or colder freezer and sent for analysis. These measurements were conducted for both groups at baseline and week 12, along with the tri-axial accelerometer (AX3 Axivity, UK), which was worn for seven consecutive days both at baseline and at week 12, similar to previous studies [28, 29]. The IN group wore the accelerometer once more, 4-weeks after the week 12 measurements (i.e., 16 weeks from baseline). The devices were programmed to record data at the prespecified start and finish times, and average weekly PA outcome measures during time awake were calculated.

A body composition machine (Inbody 230, Korea; with built-in height measurement tool BSM370, Korea) measured body mass in kilograms, height in centimetres, body fat percentage, and skeletal muscle mass in kilograms through bioelectrical impedance analysis. In addition, the validated WHO-5 Well-Being Index questionnaire and questionnaires that measures eating habits, frequency of food consumption, and PA [International Physical Activity Questionnaire (IPAQ)] were used. All questionnaires were completed using tablets. Further details regarding measurements and questionnaires are available in the published protocol [23].

## Primary outcome measures

The primary outcome was the change in cardio-metabolic risk factors, which included waist circumference, blood pressure, HDL cholesterol, triglycerides, and fasting glucose. These measurements were conducted for both groups at baseline and week 12.

## Secondary outcome measures

The secondary outcome was the change in the objectively measured PA four weeks after completing the intervention (measured at week 16 from baseline) for the IN group only.

### Statistical analysis

For the present study, it was estimated that 124 participants (62 participants in each arm) were required to achieve 80% power with an effect size of 0.51 based on previous studies related to PA and cardio-metabolic risk factors, to detect a significant between-group and within-group difference [30–33]. It was also planned that a further 20% more participants would be recruited because participants might drop out during the intervention. Therefore, the recruitment of a total of 150 participants was anticipated. During the enrolment phase between the 28th of March 2021 and the 19th of May 2021, we recruited 130 participants who fulfilled the eligibility criteria. Additional efforts were made to attract more participants to enrol in the study through email invitations and displaying information leaflets in the building’s elevators, parking areas, and restaurants/cafes. However, upon no substantial interest from the potential participants, we decided to end the recruitment phase of the trial.

An independent statistician who was blinded to group allocation performed an intention-to-treat analysis for the trial outcomes. Baseline characteristics of participants were presented as mean ± standard deviation (SD) for continuous variables and as frequencies and percentages for categorical variables. Between-group and within-group differences at week 12 were analysed using linear mixed-effects models, adjusting for age and sex. The models included fixed effects for time, group, and their interaction, with random intercepts for participants to account for repeated measures over time. An unstructured covariance matrix was used to assess the association between repeated time points. The analyses were adjusted for baseline differences for the outcome of interest. The linear mixed model includes all the participants in the analyses; therefore, no imputation of the missing values was performed, and no adjustments for multiplicity were needed.

## Results

### Participant enrolment and baseline characteristics

**Fig. 1:**
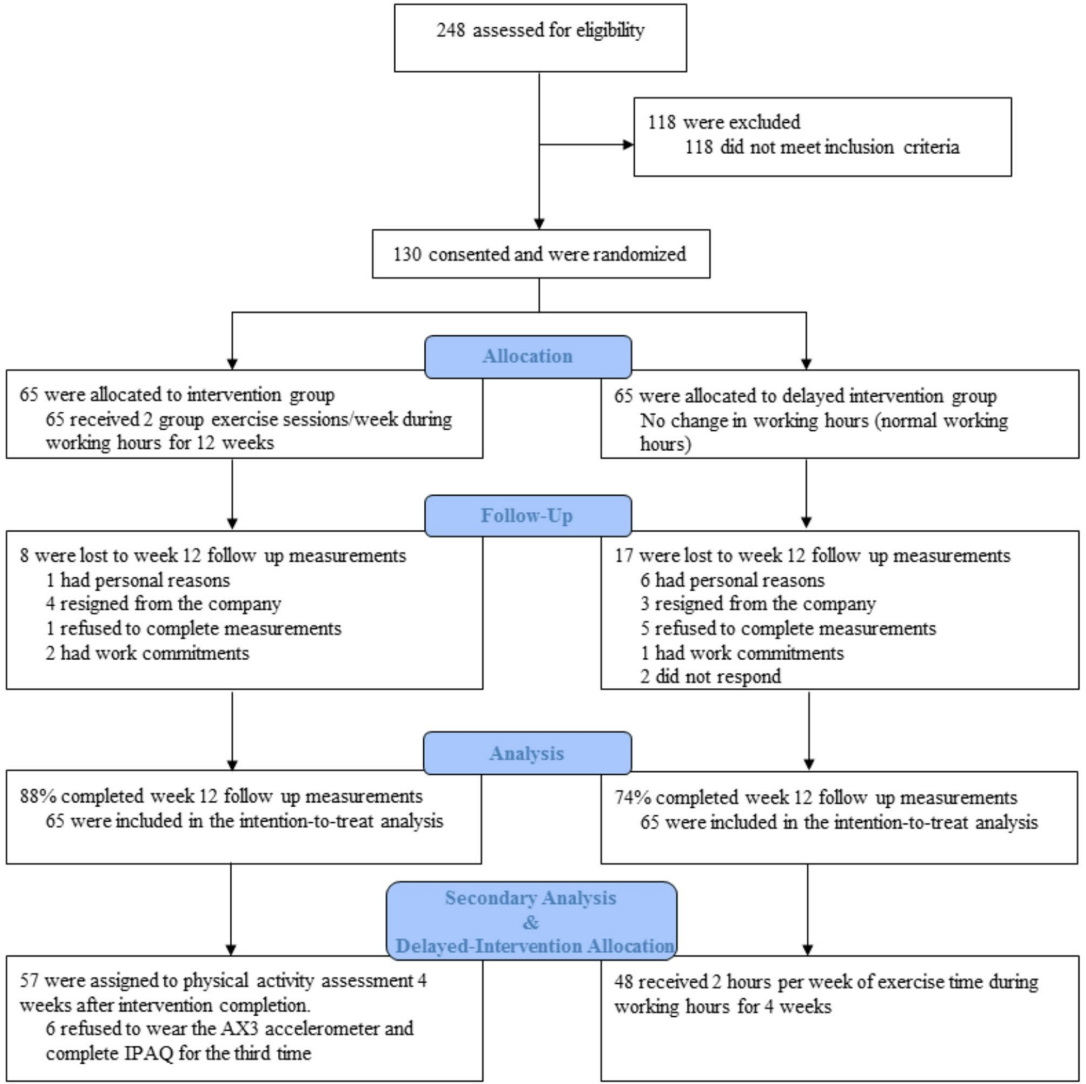
Consolidated Standards of Reporting Trials (CONSORT) Flow Diagram

Among the 2900 company employees who received the email invitation to join the study, 248 responded, of which 130 met the eligibility criteria. Figure 1 illustrates the CONSORT flow diagram. The allocation of the participants was not changed during the trial period, and there were no exclusions after the randomization process. As shown in Fig. 1, approximately 19% of the participants did not complete the post-study health measurements for various reasons. The demographic, clinical, and PA characteristics are balanced between groups, as shown in Table 1 and Table S1. The mean age was 37.3 (SD 6.6) years for IN and 36.7 (SD 6.1) years for DI group, 25% were females, and 68% had moderate to high PA scores on IPAQ. Nutritional characteristics of the participants are presented in Table S2 that were balanced between the groups except for fruit consumption.

**Table 1:** Baseline characteristics of the participants

	IN Group (*n* = 65)	DI Group (*n* = 65)
Age (years)	37.3 (6.6)	36.7 (6.1)
Males	49 (75%)	49 (75%)
Females	16 (25%)	16 (25%)
Waist circumference (cm)	102.5 (10.7)	104.2 (11.9)
Body weight (kg)	87.2 (14.9)	88.2 (17.0)
Body mass index (kg/m^2^)	29.3 (3.6)	30.0 (4.3)
Skeletal muscle mass (kg)	32.1 (7.2)	32.9 (6.8)
Body fat percentage (%)	34.7 (8.0)	34.8 (8.2)
Fasting Plasma Glucose (mg/dL)	96.3 (16.4)	99.0 (31.7)
HbA1c (%)	5.4 (0.6)	5.4 (1.0)
Total Cholesterol (mg/dL)	202.1 (40.0)	197.0 (36.7)
HDL- cholesterol (mg/dL)	45.5 (14.4)	44.9 (12.4)
LDL-cholesterol (mg/dL)	135.1 (34.4)	129.9 (31.5)
Triglycerides (mg/dL)	117.8 (59.1)	123.2 (67.3)
Systolic blood pressure (mmHg)	125.9 (12.2)	126.0 (15.7)
Diastolic blood pressure (mmHg)	82.2 (10.3)	82.0 (10.4)
Daily sitting time (hours), IPAQ	8.8 (2.7)	8.8 (2.2)
Low PA, IPAQ	19 (29%)	22 (34%)
Moderate PA, IPAQ	29 (45%)	26 (40%)
High PA, IPAQ	17 (26%)	17 (26%)
Weekly light PA (minutes), AX3	510.3 (156.9)	452.2 (124.8)
Weekly moderate PA (minutes), AX3	885.2 (293.4)	743.4 (324.8)
Weekly vigorous PA (minutes), AX3	18.8 (22.2)	20.6 (35.7)
WHO Wellbeing (score)	15.5 (4.5)	15.0 (5.1)

Values are presented as mean (standard deviation) or number of participants (%)

*IN* intervention, *DI* delayed intervention, *IPAQ* HbA1c, glycated haemoglobin, *HDL* high-density lipoprotein cholesterol, *LDL* low-density lipoprotein cholesterol, International Physical Activity Questionnaire, *WHO* World Health Organization

### Exercise session adherence

During the 12 weeks of intervention period, participants were provided with 24 one-hour exercise sessions. The adherence to exercise sessions was low, and only 14% of the participants attended 70% or more allocated sessions. Dichotomizing by the median number of exercise sessions, 45% of participants attended more than the 50th percentile (6–24 sessions), as shown in Table S3.

### Primary outcome

The main findings of the study are presented in Table 2 and Table S4. For the primary outcome, there was no statistically significant difference in cardio-metabolic risk factors between the two groups at the end of 12 weeks after adjustment of age and sex and including time and groups in the model. Table 2 presents between-group and within-group differences at baseline and 12-weeks, and overall test for interaction (time and group). The within-group mean change at 12-week was statistically significant for fasting plasma glucose [− 3.6 mg/dL (95% CI, − 6.7 to − 0.42)], HDL cholesterol [2.6 mg/dL (95% CI, 1.1 to 4.0)], and waist circumference [− 4.8 cm (95% CI, − 6.1 to − 3.5)] for the IN group. The DI group also had a significant decrease in the waist circumference [− 3.7 cm (95% CI, − 5.1 to − 2.3)] at the end of the intervention period (Table 2).

To assess whether the level of adherence influenced the outcomes, we conducted a sensitivity analysis, additionally adjusting for the number of exercise sessions attended (a proxy for intervention dose) (Table S4, Model 3). For the primary cardio-metabolic outcomes, adjusting for the received dose did not materially alter the results, and the within-group improvements over time remained significant for waist circumference, HDL cholesterol, skeletal muscle mass, and percent body fat, while the between-group differences compared to the control remained non-significant.

**Table 2:** Between-group and within-groups difference at week 12

	Within-group differences		Between-group differences	*P*-value for interaction
Measurement	IN Group	DI Group		
Fasting Plasma Glucose (mg/dL)				
Baseline value	96.3 (92.3 to 100.4)	99.0 (92.9 to 105.2)	−2.7 (− 11.4 to 6.0)	
Difference at 12 weeks	−3.6 (− 6.7 to − 0.42)	−0.4 (− 3.9 to 3.0)	−5.9 (− 14.8 to 3.1)	0.19
HbA1c (%)				
Baseline	5.4 (5.2 to 5.6)	5.4 (5.2 to 5.7)	−0.1 (− 0.4 to 0.2)	
Difference at 12 weeks	0.3 (0.2 to 0.4)	0.3 (0.2 to 0.4)	−0.1 (− 0.3 to 0.3)	0.77
Total Cholesterol (mg/dL)				
Baseline	202.1 (192.9 to 211.3)	197.0 (187.8 to 206.3)	5.1 (− 8.0 to 18.1)	
Difference at 12 weeks	−3.2 (− 10.4 to 4.0)	3.8 (− 4.0 to 11.6)	−1.9 (− 15.7 to 11.9)	0.20
HDL Cholesterol (mg/dL)				
Baseline	45.5 (43.0 to 48.1)	44.9 (42.4 to 47.5)	0.5 (− 3.1 to 4.2)	
Difference at 12 weeks	2.6 (1.1 to 4.0)	2.5 (0.9 to 4.1)	0.6 (− 3.2 to 4.3)	0.98
LDL Cholesterol (mg/dL)				
Baseline	135.1 (127.4 to 142.8)	129.9 (122.1 to 137.5)	5.3 (− 5.6 to 16.2)	
Difference at 12 weeks	−5.4 (− 11.3 to 0.5)	2.5 (− 3.9 to 8.8)	−2.6 (− 14.1 to 8.9)	0.07
Triglycerides (mg/dL)				
Baseline	117.8 (103.5 to 132.2)	123.2 (108.8 to 137.6)	−5.4 (− 25.7 to 15.0)	
Difference at 12 weeks	−1.4 (− 14.1 to 11.2)	8.1 (− 5.5 to 21.7)	−14.9 (− 36.6 to 6.9)	0.32
Waist Circumference (cm)				
Baseline	102.5 (100.3 to 104.8)	104.2 (102.0 to 106.5)	−1.7 (− 4.8 to 1.5)	
Difference at 12 weeks	−4.8 (− 6.1 to − 3.5)	−3.7 (− 5.1 to − 2.3)	−2.8 (− 6.1 to 0.5)	0.25
Body weight (kg)				
Baseline	87.2 (83.8 to 90.5)	88.2 (84.9 to 91.6)	−1.0 (− 5.8 to 3.7)	
Difference at 12 weeks	−0.1 (− 1.2 to 0.9)	1.0 (− 0.1 to 2.2)	−2.2 (− 6.9 to 2.6)	0.15
Body mass index (kg.m^−2^)				
Baseline	29.3 (28.4 to 30.3)	30.0 (29.0 to 31.0)	−0.7 (− 2.0 to 0.7)	
Difference at 12 weeks	−0.1 (− 0.3 to 0.2)	0.2 (− 0.1 to 0.5)	−0.9 (− 2.3 to 0.5)	0.24
Skeletal Muscle Mass (kg)				
Baseline	32.1 (31.0 to 33.2)	32.9 (31.8 to 34.0)	−0.8 (− 2.4 to 0.7)	
Difference at 12 weeks	0.4 (0.1 to 1.0)	0.7 (0.1 to 1.3)	−1.1 (− 2.7 to 0.5)	0.52
Body Fat Percentage (%)				
Baseline	34.7 (33.1 to 36.3)	34.8 (33.3 to 36.4)	−0.1 (− 2.4 to 2.1)	
Difference at 12 weeks	−1.0 (− 1.9 to − 0.1)	−0.8 (− 1.8 to 0.2)	−0.3 (− 2.6 to 2.0)	0.83
Systolic blood pressure (mmHg)				
Baseline	125.9 (122.8 to 129.0)	126.0 (122.9 to 129.1)	0.0 (− 4.4 to 4.4)	
Difference at 12 weeks	−2.2 (− 5.7 to 1.3)	−0.7 (− 4.4 to 3.0)	−1.5 (− 6.4 to 3.3)	0.56
Diastolic blood pressure (mmHg)				
Baseline	82.2 (79.6 to 84.5)	82.0 (79.9 to 84.5)	0.0 (− 3.3 to 3.3)	
Difference at 12 weeks	0.8 (− 1.7 to 3.3)	0.9 (− 1.8 to 3.6)	−0.1 (− 3.7 to 3.5)	0.95
Daily sitting Time (hours) –IPAQ				
Baseline	8.8 (8.2 to 9.5)	8.8 (8.2 to 9.4)	0.0 (− 0.9 to 0.9)	
Difference at 12 weeks	−0.6 (− 1.3 to 0.2)	−0.2 (− 1.0 to 0.5)	−0.3 (− 1.3 to 0.6)	0.56
WHO Wellbeing (score)				
Baseline	15.5 (14.4 to 16.6)	15.0 (13.9 to 16.1)	0.5 (− 1.1 to 2.0)	
Difference at 12 weeks	3.0 (1.8 to 4.2)	0.5 (− 0.8 to 1.8)	3.0 (1.3 to 4.7)	< 0.01
Daily sitting Time (hours) – AX3 Accelerometer				
Baseline	13.4 (13.0 to 13.9)	12.9 (12.57 to 13.4)	0.5 (− 0.1 to 1.1)	
Difference at 12 weeks	−0.2 (− 0.7 to 0.2)	0.1 (− 0.4 to 0.5)	0.2 (− 0.4 to 0.9)	0.38
Daily sleep (hours) – AX3 Accelerometer				
Baseline	6.6 (3.2 to 7.1)	7.7 (7.2 to 8.1)	−1.0 (− 1.7 to − 0.4)	
Difference at 12 weeks	0.4 (− 0.1 to 0.8)	−0.2 (− 0.7 to 0.3)	−0.5 (0.4 to 0.2)	0.09
Weekly Light Physical Activity (minutes) -AX3 Accelerometer				
Baseline	510.3 (476.1 to 544.5)	452.2 (417.9 to 486.5)	58.1 (9.7 to 106.6)	
Difference at 12 weeks	−13.7 (− 42.5 to 15.2)	−4.1 (− 35.2 to 27.0)	48.6 (− 3.0 to 100.1)	0.66
Weekly Moderate Physical Activity (minutes) - AX3 Accelerometer				
Baseline	885.2 (812.1 to 958.4)	743.4 (670.1 to 816.7)	141.9 (38.3 to 245.5)	
Difference at 12 weeks	−33.0 (− 81.0 to 14.6)	−16.5 (− 68.4 to 35.4)	125.4 (17.4 to 233.3)	0.65
Weekly Vigorous Physical Activity (minutes) - AX3 Accelerometer				
Baseline	18.8 (11.7 to 25.9)	20.6 (13.5 to 27.7)	−1.8 (− 11.8 to 8.2)	
Difference at 12 weeks	8.2 (0.4 to 15.9)	−1.2 (− 954 to 7.1)	7.5 (− 3.4 to 18.5)	0.11

Data are estimated mean (95% confidence intervals), and model estimates are after adjustments of age and sex. The overall test for interactions includes group and time

### Secondary outcome

**Fig. 2:**
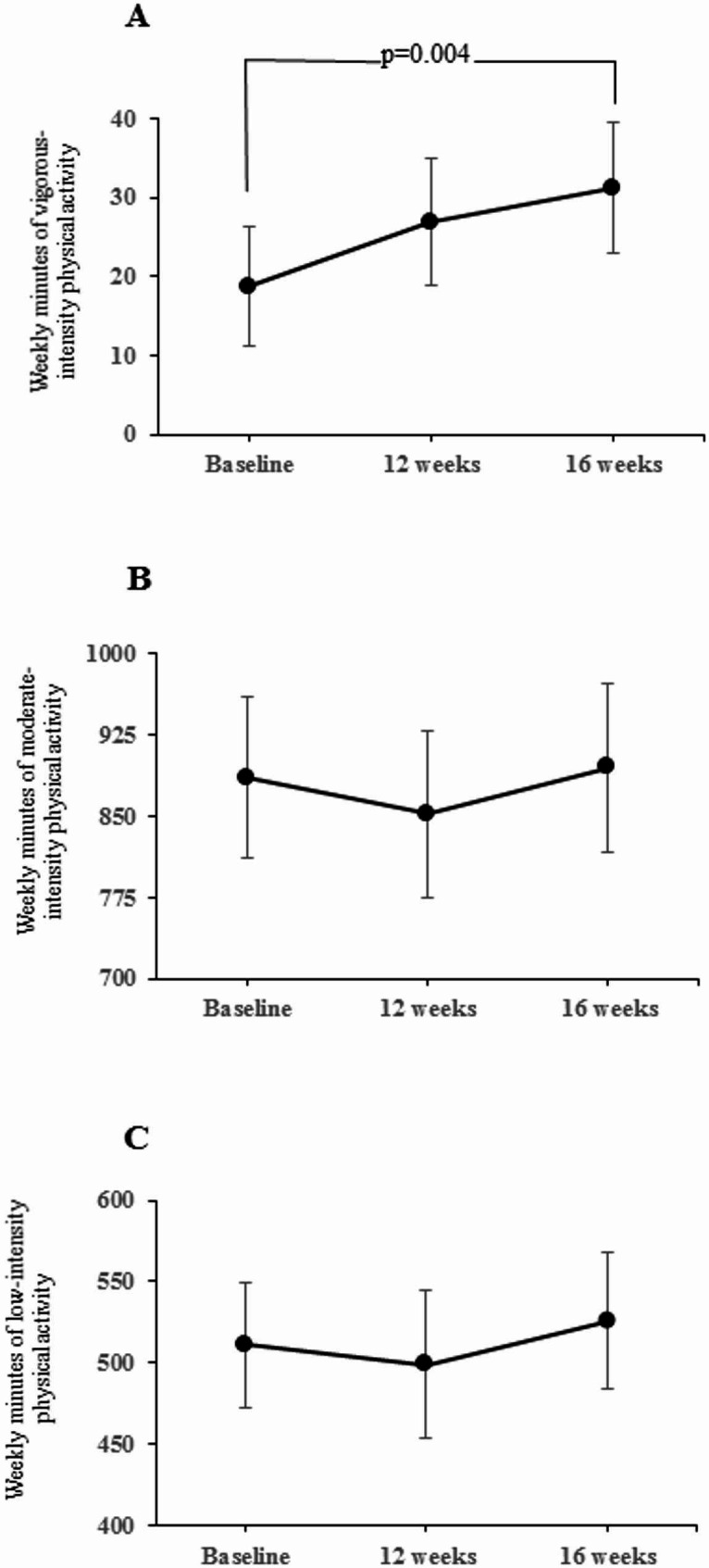
Changes in the secondary outcome of physical activity

For the secondary outcomes related to accelerometer measured PA; light, moderate, and vigorous PA increased after 4 weeks of intervention period compared to the baseline values. However, only changes in vigorous PA were significant [estimated weekly average during time awake, 12.5 min (95% CI, 4.1 to 21.0)], as presented in Fig. 2 and Table S5. The comparison between week 12 and week 16 was not significant for any exercise intensity.

### Other end points

The WHO Wellbeing score showed a statistically significant improvement at week 12 measurements compared with baseline values with a between group difference of 3.0 (95% CI, 1.3 to 4.7) (Table 2). Other estimates for between-group differences at week 12 also showed a favourable intervention effect, however, for many of these estimates, statistical significance was not achieved. Table 2 also presents statistically significant mean changes at 12-weeks for the IN group (within-group differences), such as, HDL cholesterol [2.6 mg/dL (95% CI, 1.1 to 4.0)], skeletal muscle mass [0.4 kg (95% CI, 0.1 to 1.0)], body fat percentage [− 1.0% (95% CI, − 1.9 to − 0.1)], and WHO Wellbeing score [3.0 (95% CI, 1.8 to 4.2)]. The DI group also had a statistically significant mean change at 12-week for HDL cholesterol [2.5 mg/dL (95% CI, 0.9 to 4.1)], and skeletal muscle mass [0.7 kg (95% CI, 0.1 to 1.3)].

### Adverse events

One participant in the IN group reported back pain during the first exercise session. After further investigation, it was found that the participant’s back pain existed before joining the intervention and exercise trainers took extra care to avoid any injuries. There were no further issues reported after this incident.

## Discussion

The results of our study show no statistically significant change for primary outcome between groups from baseline to the end of the 12 weeks. However, employees who were allocated time for physical exercise during their working hours improved their cardiometabolic health outcomes with significant improvements in waist circumference, fasting plasma glucose, HDL cholesterol, skeletal muscle mass, and body fat percentage. For the secondary outcomes at week 16, overall levels of PA increased with a significant improvement in the vigorous-intensity PA.

Previous studies investigating the effect of workplace exercise interventions have reported mixed results [15, 16]. Our findings related to the primary outcomes are consistent with earlier reports where no significant between-group differences for body composition and metabolic functions were reported [14–16]. In a randomized controlled trial of overweight or obese workers who were assigned to either using a treadmill workstation or working at sit-stand desks, there were no statistically significant between-group changes in BMI, waist circumference, systolic or diastolic blood pressure, fasting glucose and cholesterol levels at both 6 months or 13 months observations [14]. Similarly, the findings of a meta-analysis suggest that there is no statistically significant difference in weight loss between the intervention and control groups who were randomized for mHealth interventions at workplaces [16]. However, a positive intervention effect has also been reported with increased loss of body fat mass, percentage fat, and visceral fat level [15, 17, 18]. A small randomized study using workstation pedal desks showed reduction in visceral adipose tissue, and a moderate decrease in fasting blood glucose in the intervention group compared with the control group [18].

The inconsistencies in the findings of exercise randomized studies in workplace may be due to the differences in sample sizes, intervention types, or adherence to the intervention. For instance, in a randomized workplace study where participants were able to monitor their PA through wearable devices coupled with coaching sessions, they were significantly more likely to increase the step count and improve body composition and metabolic outcomes compared with the control group [17]. Another key factor is the adherence to the intervention in the exercise trials where low adherence may suggest that comparison groups had similar activity levels and intervention was not tested as intended [34]. In our study, intervention fidelity was low, with only 14% of participants in the intervention group attended 70% or more of the allocated sessions despite all the efforts to retain attendance and compliance. Earlier results of personalized behaviour intervention studies also show higher withdrawal or dropout rates [15], and factors associated with non-adherence to exercise intervention have been extensively studied [35, 36]. These factors include higher baseline BMI, low baseline fitness levels, a combination of lack of time due to family and/or work, motivation, societal and social pressures, and lack of enjoyment of exercise [36–39]. For example, the STRRIDE trials found that lack of time, motivation, and physical limitations were the most frequent reasons for attrition in structured exercise interventions [36]. In our study, competing work demands and persistent time pressures likely represented major barriers which may have persisted despite the allocated exercise time, along with insufficient intrinsic motivation for regular, structured exercise among some participants. Furthermore, the results of the sensitivity analyses additionally adjusting for the number of exercise sessions attended demonstrated that session attendance alone may not be the sole determinant of the outcome. Recent meta-analytical evidence also showed a negligible or inconsistent direct association between the number of intervention sessions and effectiveness in PA trials [40–42]. This suggest that other factors beyond simple attendance, such as the Hawthorne effect, increased health awareness, or changes in behaviour outside of the supervised sessions [43, 44], may have contributed to the within-group improvements observed among participants, regardless of their adherence level.

Another possible reason for the low attendance in the exercise sessions in our study could be the COVID-19 pandemic during the intervention period (May to August 2021). For example, in May 2021, WHO designated the Delta variant as a variant of concern [45]. In addition, the weekly COVID-19 cases remained relatively high in the United Arab Emirates during the intervention period [46]. The trial investigators informed the participants that all safety measures were applied (e.g., a maximum of 10 participants per session, physical distancing of more than one meter, and sanitization of equipment with Isopropyl Alcohol 70% solution after every session, among others).

Throughout the intervention period of 12 weeks, there was one positive COVID-19 case. The exercise trainer and other participants in the same exercise session were informed and requested to follow the close-contact tracing process, and were asked to perform a PCR test before their next session.

The observed null findings of our trial must be interpreted in the context of statistical power. Post-hoc calculations show that with the achieved sample (*n* = 57 intervention, *n* = 48 control) and observed variability, the trial had 80% power to detect differences of 3.8 cm in waist circumference, 4.9 mmHg in systolic blood pressure, 8.9 mg/dL in fasting plasma glucose, and 11.5 mg/dL in LDL-cholesterol. While the intervention did not yield statistically significant results, we cannot rule out the possibility of a smaller, yet clinically meaningful, positive effect that our study was not designed to detect.

The accelerometer measured data showed that the study population was profoundly sedentary, averaging approximately 13 h of daily sitting time at baseline. This is consistent with their roles in an office-based workplace, which inherently involves prolonged sitting. Our intervention was not designed to specifically target or reduce the extensive sedentary time that characterized participants’ days. Nonetheless, other workplace trials such as the one by Bergman et al. found that treadmill workstations did not significantly reduce daily sitting time over a 13 months intervention period [14]. Even though, an increase in the moderate-to-vigorous PA was observed in our study, it was likely insufficient to overcome the cardiometabolic burden of prolonged sedentary time [47]. Our results contribute to a complex body of literature suggesting that for highly sedentary populations, the most beneficial approach requires integrated strategies that simultaneously increase PA and reduce sedentary behavior [7, 48]. Future workplace interventions should combine structured exercise with environmental changes (e.g., sit-stand desks) and behavioural prompts (e.g., break reminders) to effectively disrupt and reduce prolonged sitting [7, 48–50].

### Strengths and limitations

We conducted a pragmatic, single-blinded randomized controlled trial. The study procedures are in alignment with the ENWHP recommendations and SEM. For example, the intervention provided accessible and motivational PA sessions during working hours. In addition, the study reflected upon different SEM levels, such as the individual, interpersonal, organizational, and policy/enabling environment levels during the planning phase. Another important aspect of the study is the objective measurements (AX3 Accelerometer) for PA alongside the subjective measurements (IPAQ). The accelerometers used in this study measured PA at three different time points for the IN group: baseline, post-study, and 4-weeks post-study. The third measurement (4-weeks post-study) assessed whether the intervention effect on PA remained 4 weeks after the trial end period. This data is important to reflect upon the sustained and long-term benefits of exercise.

For our trial, recruitment was open for all working ages (e.g., 18–59), both sexes, those with office or non-office job positions, and all nationalities. The broad spectrum of characteristics allowed the trial to be representative of workplaces with similar settings (e.g., 9 am to 5 pm working hours and availability of a workplace gym). All the eligible participants agreed to participate in the trial, indicating a favourable preference and acceptability for the intervention. Therefore, it is reasonable to assume that the benefits of the trial could be generalizable to workplaces with similar settings. Even though participants were strictly advised not to discuss their allocated group, the risk of contamination remained. For example, some participants were colleagues or were friends and therefore may have been able to discern group allocation.

Furthermore, our study did not examine participants’ medication status, which could potentially impact their cardiometabolic health outcomes. In a 2018 meta-analysis of 28 trials, weight-loss medications showed modest reductions in fasting blood glucose − 4.0 mg/dL (95% CI, − 4.4 to − 3.6) and waist circumference − 3.3 cm (95% CI, − 3.5 to − 3.1) [51]. Effects varied among drugs (e.g., Phentermine-topiramate, Liraglutide, Naltrexone-bupropion), but none improved all outcomes. Given the relatively healthy profile of participants in our study, it is unlikely that these participants would be on any weight-loss medications. However, this generally healthy profile of our participants at baseline may also have limited the potential for significant improvement. Although, participants were recruited based on their baseline elevated waist circumference, the baseline mean values of several key cardio-metabolic risk factors were close to the normal clinical range. This ceiling effect likely reduced the potential to detect a significant intervention effect compared to a trial recruiting a more clinically at-risk population.

The low intervention fidelity as evidenced by the poor attendance rate is a key limitation of our trial, impacting the interpretation of the effectiveness of the intervention and its generalizability. However, this issue is prevalent in pragmatic lifestyle interventions, where standard engagement strategies can fail for a substantial proportion of participants [36]. Our observations highlight that providing access and time to exercise during working hours is important but must also directly target motivation and habit formation to have better outcomes. Future workplace exercise studies should implement and test multifaceted retention strategies informed by behavioural science, integrating tailored behaviour change techniques including goal setting, self-monitoring, action planning, and motivational interviewing to strengthen intrinsic motivation and engagement [52, 53]. Furthermore, providing accessible and enjoyable exercise options, and building in peer and social support can address diverse participant preferences and strengthen adherence. The use of digital health tools for reminders, feedback, efficient tracking, personalized communication, and appropriate incentives can help to reduce practical barriers, and may enhance retention in PA programs [54, 55].

## Conclusion

In this pragmatic, randomized controlled trial, we found no statistically significant between-group differences for the cardio-metabolic risk factors at the end of 12 weeks of exercise intervention. However, the intervention group showed promising within-group improvements in specific cardio-metabolic factors, and maintained increased PA levels four weeks post intervention. Future large-scale studies are needed to investigate the effectiveness of exercise interventions in different workplace settings and working conditions to confirm the potential cardiometabolic benefits, and to evaluate the impact of such programs on organizational outcomes like productivity to incentivize broader implementation.

## Supplementary Information

Supplementary Material 1.

Supplementary Material 2.

## Data Availability

The datasets used and analyzed during the current study are available from the corresponding author on reasonable request.

## Abbreviations

BMI: Body Mass Index
DBP: Diastolic Blood Pressure
DI: Delayed-Intervention Group
ENWHP: European Network for Workplace Health Promotion
FPG: Fasting Plasma Glucose
HDL: High-Density Lipoprotein
IN: Intervention Group
IPAQ: International Physical Activity Questionnaire levels
LDL: Low-Density Lipoprotein
LPA: Low Physical Activity
MPA: Moderate Physical Activity
PA: Physical Activity
PBF: Percentage of Body Fat
TC: Total Cholesterol
SBP: Systolic Blood Pressure
SD: Standard Deviation
SEM: Social-Ecological Model
SMM: Skeletal Muscle Mass
Trig: Triglycerides
VPA: Vigorous Physical Activity
WC: Waist Circumference
WCF: Waist Circumference Females
WCM: Waist Circumference Males
WHO: World Health Organization
