# Supplementary material for: Effects of a workplace exercise intervention on cardiometabolic health: a randomized controlled trial
Source: BMC Public Health. 2025 Oct 15;25:3493. doi: 10.1186/s12889-025-24815-5 (PMC12522563; doi:10.1186/s12889-025-24815-5)
Supplement: Supplementary file 2 — Supplementary Material 2. [file 12889_2025_24815_MOESM2_ESM.pdf]

Supplementary Materials

EFFECTS OF A WORKPLACE EXERCISE INTERVENTION ON CARDIOMETABOLIC HEALTH: RANDOMIZED CONTROLLED TRIAL

Ali Muneer Al Rahma, Mansoor Anwar Habib, Emad Masuadi, Thomas Boillat, Syed Mahboob Shah, Luai Awad Ahmed, Tom Loney, Javaid Nauman

Published Study Protocol

Previously published in BMJ Open:

Alrahma AM, Habib MA, Oulhaj A, et al. Effects of a workplace exercise intervention on cardiometabolic health: study protocol for a randomised controlled trial. BMJ Open 2021;11(11):e051070. Available open access here:

https://bmjopen.bmj.com/content/11/11/e051070

Table S1: Baseline characteristics of participants

	**IN Group (n=65)**	**DI Group (n=65)**	***P***
**Age (years)**	37.3 (6.6)	36.7 (6.1)	0.55
**Nationality**			0.66
Indian	27 (41%)	30 (46%)	
Pakistani	12 (18%)	13 (20%)	
Filipino	3 (5%)	6 (9%)	
Emirati	3 (5%)	1 (2%)	
Other Nationalities	20 (31%)	15 (23%)	
**Physical Activity Category (IPAQ)**			0.82
Low	19 (29%)	22 (34%)	
Moderate	29 (45%)	26 (40%)	
Vigorous	17 (26%)	17 (26%)	
**Cardio-metabolic Risk Factors**			
Elevated Waist circumference (cm)	65 (100%)	65 (100%)	-
Elevated Blood Pressure (mmHg)	34 (52%)	38 (58%)	0.48
Reduced HDL- cholesterol (mg/dL)	34 (52%)	28 (43%)	0.29
Elevated Triglycerides (mg/dL)	18 (28%)	16 (25%)	0.69
Elevated Fasting glucose (mg/dL)	14 (22%)	13 (20%)	0.82
**Number of Meals per Day**			0.39
1-2 Meals	31 (48%)	27 (41%)	
3-4 Meals	31 (48%)	31 (48%)	
5 or More Meals	3 (4%)	7 (11%)	
**Skipping Meals**			0.37
Yes	36 (55%)	41 (63%)	
No	29 (45%)	24 (37%)	
**Reason for Skipping Meals (if yes)**			0.60
To Reduce Food Intake	6 (17%)	9 (22%)	
To Lose Weight	19 (52%)	19 (46%)	
Lack of Appetite	5 (14%)	9 (22%)	
Fasting	6 (17%)	4 (10%)	
**Water Consumed per Day**			0.78
1-4 Cups	14 (22%)	16 (25%)	
5-7 Cups	34 (52%)	30 (46%)	
8 or More Cups	17 (26%)	19 (29%)	

Values are presented as mean (standard deviation) or number of participants (%).
IPAQ, International Physical Activity Questionnaire; IN, intervention; DI, delayed intervention.

Table S2: Baseline nutrition characteristics

Intervention Group (n=65)					
**Frequency of Food Consumption**	**4 or more/day**	**2-3 times/day**	**Once/day**	**1-4 times/week**	**Never**
Fruits*	0	15 (23%)	33 (51%)	13 (20%)	4 (6%)
Vegetables	0	22 (34%)	24 (37%)	19 (29%)	0
Milk and milk products	1 (2%)	16 (25%)	23 (35%)	21 (32%)	4 (6%)
Meat/fish/chicken	2 (3%)	10 (16%)	23 (35%)	24 (37%)	6 (9%)
Bread/rice/pasta	1 (2%)	15 (23%)	24 (37%)	23 (35%)	2 (3%)
Sweets/desserts	2 (3%)	8 (12%)	15 (23%)	33 (51%)	7 (11%)
Salty snacks	1 (2%)	9 (14%)	20 (30%)	28 (43%)	7 (11%)
Coffee/tea	8 (12%)	30 (46%)	19 (29%)	7 (11%)	1 (2%)
Sweetened drinks	1 (2%)	1 (2%)	9 (14%)	32 (49%)	22 (33%)
Energy drinks	0	0	3 (5%)	8 (12%)	54 (83%)
**Delayed-Intervention Group (n=65)**					
**Frequency of Food Consumption**	**4 or more/day**	**2-3 times/day**	**Once /day**	**1-4 times/week**	**Never**
Fruits	0	9 (14%)	25 (38%)	29 (45%)	2 (3%)
Vegetables	0	19 (29%)	21 (32%)	24 (37%)	1 (2%)
Milk and milk products	2 (3%)	13 (20%)	25 (38%)	20 (31%)	5 (8%)
Meat/fish/chicken	1 (2%)	16 (24%)	27 (42%)	16 (24%)	5 (8%)
Bread/rice/pasta	1 (2%)	30 (46%)	20 (31%)	12 (18%)	2 (3%)
Sweets/desserts	2 (3%)	7 (11%)	17 (26%)	34 (52%)	5 (8%)
Salty snacks	1 (2%)	9 (14%)	18 (28%)	31 (47%)	6 (9%)
Coffee/tea	5 (8%)	36 (55%)	13 (20%)	5 (8%)	6 (9%)
Sweetened drinks	1 (2%)	3 (5%)	6 (9%)	33 (51%)	22 (33%)
Energy drinks	1 (2%)	0	2 (3%)	9 (14%)	53 (81%)

Values are presented as the number of participants (%). *Statistically significant chi-square test.

Table S3: Number of exercise sessions attended

Percentile	Number of participants	Exercise sessions
51%-100%	29 (45%)	6-24
0-50%	36 (55%)	0-5
≥70%	9 (14%)	16-24

Table S4: Linear mixed models output using outcome data at week 12

Measurement	Model 1			Model 2			Model 3		
	B0	B1	B2	B0	B1	B2	B0	B1	B2
**HbA1c**	5.41	0.35*	-0.04	5.39	0.35*	-0.04	5.37	0.34*	-0.03
**FPG**	98.38	-1.54	-1.62	79.72	-1.55	-1.94	78.1	-1.56	-0.32
**TC**	196.42	0.07	2.25	193.2	0.05	2.24	191.24	0.08	4.24
**HDL**	44.73	2.6*	0.86	39.3	2.57*	0.55	40.01	2.56*	-0.18
**LDL**	131.56	-1.77	1.86	128.82	-1.77	2.08	126.91	-1.74	4.02
**Trig**	125.61	2.93	-10.07	122.07	2.99	-9.59	120.4	3.03	-7.86
**WC**	104.33	-4.25*	-2.05	93.65	-4.27*	-2.03	93.53	-4.27*	-1.9
**WCF**	93.63	-4.3*	-2.67	94.95	-4.3*	-2.46	92.82	-4.3*	-0.15
**WCM**	107.55	-4.23*	-1.99	102.99	-4.23*	-1.97	103.94	-4.23*	-2.74
**SBP**	125.99	-1.3	-0.37	103.84	-1.48	-0.71	103.58	-1.48	-0.44
**DBP**	82.03	0.94	0.16	71.32	0.86	-0.05	70.45	0.88	0.86
**Weight**	88.57	0.39	-1.74	80.99	0.39	-1.45	80.1	0.39	-0.61
**BMI**	30	0.09	-0.66	30.38	0.09	-0.62	30.58	0.09	-0.82
**SMM**	32.92	0.56*	-0.94	23.17	0.55*	-0.95	22.07	0.56*	0.17
**PBF**	35.0	-0.94*	-0.35	45.82	-0.95*	-0.28	46.88	-0.96*	-1.5
**Sitting, IPAQ**	8.91	-0.43	-0.14	9.56	-0.42	-0.29	9.60	-0.42	-0.17
**Sleep, weekly minutes**	2696.8	26.66	-257.7*	2387.12	23.49	-262.2*	2240.88	27.05	-108.96
**Sitting, weekly minutes**	4660.85	-155.1	180.95	4824.61	-149.3	185.42	4875.63	-156.8	137.14
**LPA, weekly minutes**	453.89	-9.48	55.0*	485.5	-9.40	54.77*	504.72	-9.68	34.86
**MPA, weekly minutes**	749.35	-26.03	132.62*	965.58	-25.51	135.34*	999.37	-25.93	97.11
**VPA, weekly minutes**	18.69	3.76	2.15	24.66	3.82	2.33	21.51	3.94	5.64
**Wellbeing**	14.35	1.87*	1.82*	12.05	1.56*	1.80*	13.46	1.83*	0.73

Model 1: including time and groups.
Model 2: including time, groups, age, and sex.
Model 3: including time, groups, age, sex, and exercise (number of sessions).
B0: the measurement at baseline for the control group
B1: the measurement difference between baseline and 12 weeks (12 week – baseline).
B2: the measurement difference between intervention and control group (Intervention – control).
**P*-value <0.05

Table S5: Within-group differences at weeks 12 and 16 compared with baseline measurements for the intervention Group (physical activity measurements only).

**Measurement**	**Intervention Group**
Baseline weekly light physical activity (minutes) – AX3	510.3 (476.1 to 544.5)
Difference at 12 weeks	−13.7 (−42.5 to 15.2)
Difference at 16 weeks	5.3 (−24.4 to 35.0)
Difference 12 vs 16 weeks	18.9 (−11.0 to 48.9)
Baseline weekly moderate physical activity (minutes) – AX3	885.2 (812.1 to 958.4)
Difference at 12 weeks	−33.0 (−81.0 to 14.6)
Difference at 16 weeks	10.4 (−40.9 to 61.8)
Difference 12 vs 16 weeks	43.3 (−8.4 to 94.9)
Baseline weekly vigorous physical activity (minutes) – AX3	18.8 (11.7 to 25.9)
Difference at 12 weeks	8.2 (0.4 to 15.9)
Difference at 16 weeks	12.5 (4.1 to 21.0)
Difference 12 vs 16 weeks	4.3 (−4.2 to 12.8)
